# Oxidative stress, free radicals and antioxidants: potential crosstalk in the pathophysiology of human diseases

**DOI:** 10.3389/fchem.2023.1158198

**Published:** 2023-05-10

**Authors:** Priya Chaudhary, Pracheta Janmeda, Anca Oana Docea, Balakyz Yeskaliyeva, Ahmad Faizal Abdull Razis, Babagana Modu, Daniela Calina, Javad Sharifi-Rad

**Affiliations:** ^1^ Department of Bioscience and Biotechnology, Banasthali University Vanasthali, Rajasthan, India; ^2^ Department of Toxicology, University of Medicine and Pharmacy of Craiova, Craiova, Romania; ^3^ Al-Farabi Kazakh National University, Faculty of Chemistry and Chemical Technology, Almaty, Kazakhstan; ^4^ Department of Food Science, Faculty of Food` Science and Technology, Universiti Putra Malaysia, Selangor, Malaysia; ^5^ Natural Medicines and Products Research Laboratory, Institute of Bioscience, Universiti Putra Malaysia, Selangor, Malaysia; ^6^ Department of Biochemistry, Faculty of Science, University of Maiduguri, Maiduguri, Nigeria; ^7^ Department of Clinical Pharmacy, University of Medicine and Pharmacy of Craiova, Craiova, Romania; ^8^ Facultad de Medicina, Universidad del Azuay, Cuenca, Ecuador

**Keywords:** free radicals, oxidative stress, pathophysiology, chronic diseases, phytochemicals, antioxidants

## Abstract

**Introduction:** Free radicals are reactive oxygen species that constantly circulate through the body and occur as a side effect of many reactions that take place in the human body. Under normal conditions, they are removed from the body by antioxidant processes. If these natural mechanisms are disrupted, radicals accumulate in excess and contribute to the development of many diseases.

**Methodology:** Relevant recent information on oxidative stress, free radicals, reactive oxidative species, and natural and synthetic antioxidants was collected by researching electronic databases such as PubMed / Medline, Web of Science, and Science Direct.

**Results:** According to the analysed studies, this comprehensive review provided a recent update on oxidative stress, free radicals and antioxidants and their impact on the pathophysiology of human diseases.

**Discussion:** To counteract the condition of oxidative stress, synthetic antioxidants must be provided from external sources to supplement the antioxidant defense mechanism internally. Because of their therapeutic potential and natural origin, medicinal plants have been reported as the main source of natural antioxidants phytocompounds. Some non-enzymatic phytocompounds such as flavonoids, polyphenols, and glutathione, along with some vitamins have been reported to possess strong antioxidant activities in vivo and in vitro studies. Thus, the present review describes, in brief, the overview of oxidative stress-directed cellular damage and the unction of dietary antioxidants in the management of different diseases. The therapeutic limitations in correlating the antioxidant activity of foods to human health were also discussed.

## 1 Introduction

Human beings have always involved themselves in various activities to ensure their wellbeing and survival. In doing so, the human body has directed the release of different free radicals or reactive substances which are either inhaled or consumed (Engwa, 2018). The reactive nitrogen and oxygen species (RNS/ROS) play a twofold role as both toxic and beneficial compounds to the organism’s system. At lower concentrations, they have beneficial effects and indulged in different physiological processes such as redox regulation, mitogenic responses, cellular signaling pathways, and an immune function while at a higher level, these reactive species generate nitrosative and oxidative stress (Phaniendra et al., 2015). To reduce or prevent free radical-directed oxidative damage, the human body has developed an antioxidant defence mechanism that involves free radical scavenging, metal chelating, and enzymatic activities to neutralize the reactive species just after they have formed. In addition, the consumption of dietary antioxidants can maintain an adequate level of antioxidants in the organism’s body (Lobo et al., 2010). The level of reactive species in the cellular system may be reduced by antioxidants either by restricting the expression or activities of free radical-producing enzymes such as xanthine oxidase (XO) and NAD(P)H oxidase, or by enhancing the expression and activities of antioxidant enzymes such as glutathione peroxidase (GPx), catalase (CAT), and superoxide dismutase (SOD) (Aziz et al., 2019). The growing interest in antioxidants among the public, health professionals, and food scientists is due to their protective function in food items against oxidative deterioration and the organism body against oxidative stress-directed abnormal processes. These potent natural antioxidants are in huge demand for pharmaceuticals/nutraceuticals and as food preservatives. Effective search for new sources of naturally occurring antioxidants and formulation of new antioxidant compounds need reliable methods for evaluation of the antioxidant activity. Many biological models, food models, and chemical assays have been developed that can measure the reducing power, radical scavenging activity and other related attributes along with overall oxidation inhibition in the more complex biological system and food items. These processes vary in terms of ease of operation, result expression, oxidation initiator, substrate type, and antioxidant mechanism. Selection of a specific or combination of the method is required for the proper assessment of antioxidant potential as a health-enhancing agent or as a food preservative (Shahidi and Zhong, 2015; Chaudhary and Janmeda, 2022a). Thus, the current review updates the current information about free radicals, their origin, types, and antioxidants with their mode of action against reactive species. This paper is found to be very helpful for scientists and researchers who are working on the investigation of the molecular and chemical mechanism of antioxidants. It also benefits the physician who is interested in antioxidant therapy for the cure of diseases.

## 2 Methodology

Relevant recent information on oxidative stress, free radicals, reactive oxidative species, and natural and synthetic antioxidants was collected by researching electronic databases such as PubMed/Medline, Web of Science, and Science Direct using the following MeSH terms: “Antioxidants/analysis”, “Antioxidants/metabolism”, “Antioxidants/therapeutic use”, “Diet”, “Food Analysis, Forecasting, Free Radicals/adverse effects”, “Free Radicals/antagonists and inhibitors”, “Free Radicals/metabolism”, “Humans”, “Lipid Peroxidation/physiology”, “Reactive Oxygen Species/adverse effects”, “Reactive Oxygen Species/metabolism”, “Reactive Oxygen Species/therapeutic use”, “Antioxidants/metabolism Free Radicals/adverse effects”, “free Radicals/metabolism”, “Humans Oxidative Stress Oxygen/metabolism”. The taxonomy of the plants mentioned in this review was verified using “The PlantList”.

## 3 Reactive oxygen species (ROS): From physiology to pathology

Free radicals are molecular species that exist independently and contain an unpaired form of an electron in their atomic orbital. These radicals are highly unstable and reactive. They either donate or accept an electron, therefore acting as oxidants and reductants (Lobo et al., 2010). The generation of highly reactive ROS is an important feature of the normal cellular system such as fertilization, ovulation, arachidonic acid metabolism, phagocytosis, and mitochondrial respiratory chain. Their generation gets multiplies many folds during pathological complications. During the recovery phases, the release of oxygen-free radicals has been observed from many pathological stimuli and reached cerebral tissue (Snezhkina et al., 2020). Reactive species include nitric oxide (•NO), alkoxy (-OR), peroxyl (ROO·), hydroxyl (·OH), hydrogen peroxide (H_2_O_2_), and superoxide (O_2_^.-^), respectively. Majorly the superoxide radicals are generated from microsomal and mitochondrial electron transport chains. Except for cytochrome oxidase which retains the reduced form of oxygen intermediates to their active site, all the other elements of the mitochondrial respiratory chain transfer the electrons directly to oxygen and do not retain any reduced intermediate of oxygen at their active site to protect the cell against oxidative damages. On the internal membrane of mitochondria, the superoxide anions may be produced by the auto-oxidation of semiquinones. A major part of mitochondrial-generated superoxide anions is dismutation to H_2_O_2_. The alkoxyl and hydroxyl free radicals are reactive and rapidly target the major macromolecules in cells (Strzelak et al., 2018). Free radicals produced by ROS direct undesirable changes and result from lipid peroxidation, DNA fragmentation, cell death, DNA damage, protein modification, and membrane damage. This oxidative stress is not only involved in the toxicity of xenobiotics but also in the pathophysiology of various ailments like ischaemia reperfusion injury, vascular endothelium, deep injuries, organ dysfunction, shock, inflammation, sepsis, diabetic retinopathy, cancer, cognitive dysfunction, cataract, and heart disease. Alterations in the concentration of iron have been observed in amyotrophic lateral sclerosis, spastic paraplegia, and multiple sclerosis which strengthens the knowledge that the accumulation of iron is a secondary factor that is related to neurodegenerative diseases. It can also be associated with gliosis in the affected area or alter the integrity of the blood-brain barrier resulting in inflammatory events and altered tissue vascularization (Stephenson et al., 2014).

### 3.1 Roles of ROS and NO in the pathophysiology of human diseases

#### 3.1.1 Oxidative stress, ROS, inflammatory markers and different types of cancer: Connecting the dots

##### 3.1.1.1 Role in carcinogenesis

The mechanism of carcinogenesis is divided into different stages: progression, promotion, and initiation. In the initiation stage, an endogenous or exogenous carcinogen induces certain changes in the DNA of a cell. This causes an abnormal change that can be inheritable and confers that the cell has the capacity for neoplastic growth (Klaunig and Kamendulis, 2004). This is reported after the metabolic activation of a procarcinogen, which includes oxidative metabolism directed by phase I enzyme to produce reactive oxygen species and electrophiles. The potency of carcinogens is determined by the ability to induce heritable alterations in the specific gene or by the stability of adducts formed with DNA (Klaunig and Kamendulis, 2004). DNA damage is the main cause of several diseases like cancer. The main focus has been given to the consequences and extent of Dfocusations in response to oxygen radicals and other associated reactive species. ROS damaged the cellular DNA which is determined to be a main carcinogenic factor This mis-repaired DNA can result in mutations. These mutations carried out the conversion of a proto-oncogene into a carcinogenic oncogene, which is responsible for different types of cancer (Marnett, 2000). The main damage is the generation of hydroxylated bases of DNA, which is regarded as a crucial event in the process of chemical carcinogenesis (Marnett, 2000). RNS/ROS can cause damage to nucleic acid. The mitochondrial DNA is more prone to get attached by ROS as compared to the nuclear DNA because it is present close to the site of ROS generation. Hydroxyl radicals directly target the different components of DNA such as deoxyribose sugar backbone, pyrimidine and purine bases, and result from single and double-stranded breaks in the DNA (Nita and Grzybowski, 2016). The purine targeted by hydroxyl radical generates various purine adducts like 2,6-diamino-4-hydroxy-5-formamidopyrimidine, 8-hydroxy deoxy adenosine, and 8-hydroxydeoxy guanosine. The pyrimidine adducts formed by the attack of hydroxy radical include hydantoin, 5-hydroxy deoxycytidine, 5-hydroxydeoxy uridine, uracil glycol, and thymine glycol, respectively. On another side, the RNS, mainly the peroxynitrite (ONOO^−^) interact with guanine to generate oxidative and nitrative DNA damage such as 8-oxodeoxyguanosine and 8-nitroguanine, respectively (Phaniendra et al., 2015).

##### 3.1.1.2 Chronic inflammation, cancer and ROS

**FIGURE 1:**
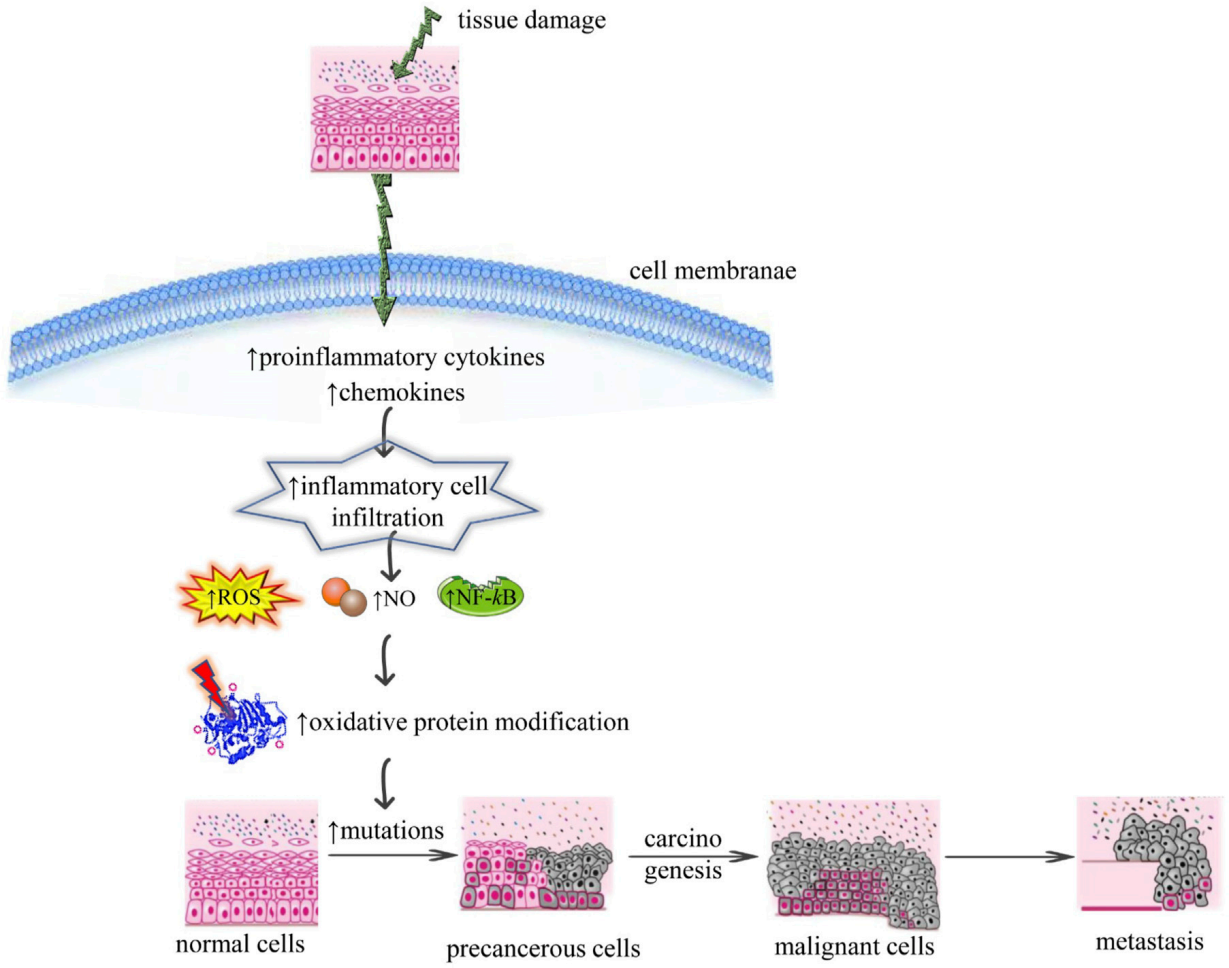
Illustrative scheme regarding the correlation between chronic inflammation and cancer mechanisms. Abbreviations and symbols: ↑increase, ↓decrease, ROS (reactive oxidative species), NO (nitric oxide), NF-*κ*B (nuclear factor kappa-light-chain-enhancer of activated B cells).

The main component in the relationship between chronic inflammation and cancer is ROS as shown in Figure 1. It can target the level, presence, and type of modulating factors related to inflammation such as growth factors, chemokines, inflammatory cytokines, p53, peroxisome proliferated-activated receptor gamma (PPAR-γ), NF-kB, hypoxia-inducible factor (HIF-1α), *ß*-catenin/Wnt (wingless related integration site), and activator protein 1 (AP-1) (Reuter et al., 2010; Kashyap et al., 2019). There is typical crosstalk between cancer progression, ROS accumulation, and chronic inflammation Under tumor microenvironment, inflammatory cells direct a massive generation of ROS by the activation of oxidant-generating enzymes such as upregulation of lipoxygenase (LOX), myeloperoxidase (MPO), cyclooxygenase 2 (COX2), xanthine oxidase (XO), NADPH oxidase, and inducible nitric oxide synthase (iNOS) to disrupt the physical, chemical, and biological factors. Excessive ROS accumulation causes oxidative damage to mitochondria, lipids, proteins, RNA, and DNA (Reuter et al., 2010; Kashyap et al., 2019).

##### 3.1.1.3 Cancer metastasis and ROS

Excessive level of ROS leads to metastasis via the stimulation of mitogen-activated protein kinase (MAPK), and phosphoinositide-3-kinase regulatory subunit/AKT serine/threonine kinases/mechanistic target of rapamycin kinase (PI2K/Akt/mTOR) signaling pathways that directs the activation of downstream metalloproteinase 9 (MMP9), metalloproteinase 2 (MMP2), SNAIL enzymes which initiate epithelial-mesenchymal transition (EMT) to metastasis (Deepika et al., 2013). The ROS results in genetic instability due to mutation load and DNA damage. Exposure to ROS can also bring modulation the expression of different transcription factors such as nuclear factor kappa B (NF-kB), and activator protein 1 (AP-1), involved in cancer stem cell maintenance, metastasis, and proliferation. It is determined that ROS are implicated in different cancer-associated processes such as angiogenesis, inflammation, metastasis, and apoptosis (Kashyap et al., 2019).

##### 3.1.1.4 Pro-angiogenic role of ROS

**FIGURE 2:**
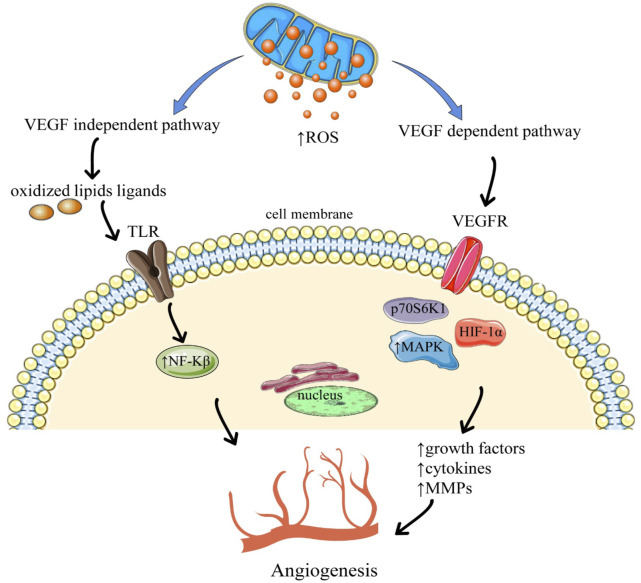
Angiogenesis through the activation of reactive oxygen species. Abbreviations and symbols: ↑ increased, ↓ decreased, ROS reactive oxygen species, TLR Toll-like receptors, VEGFR Vascular endothelial growth factors, NF-kβ nuclear factor kappa B, MAPK mitogen-activated protein kinase, HIF-1α (Hypoxia-inducible factor 1-alpha).

Activation of angiogenesis through ROS via vascular endothelial growth factor (VEGF) independent and VEGF dependent pathways are shown in Figure 2. The VEGF-dependent pathway raises the expression of vascular endothelial growth factor (VEGF) *via* MAPK (Mitogen-activated protein kinases), and the phosphoinositide-3-kinase regulatory subunit/AKT serine/threonine kinases/mechanistic target of rapamycin kinase (PI3K/Akt/mTOR), PTEN (phosphatase and tensin homolog) signaling pathway via p70S6K1 (ribosomal protein S6 kinase B1) and HIF-1α (Hypoxia-inducible factor1-alpha), that releases different growth factors, cytokines, and undergo the upregulation of MMPs which leads to angiogenesis. Whereas the VEGF-independent pathway results in angiogenesis *via* oxidative ligands of lipids which activate NF-kB *via* Toll-like receptor (TLR) (Deepika et al., 2013).

##### 3.1.1.5 Lung cancer

The lung is the only organ that is under more exposure to ambient air than the skin. It is therefore easily affected by oxidants, pathogens, pollutants, gases, and inhaled toxins. In addition, endogenous oxidants produced by different pathophysiological processes in the lung and anywhere in the body also increased the oxidative burden. To control these effects, the epithelium of the lung is protected by a lining of fluid containing an armamentarium of antioxidants. When the equilibrium between the antioxidants and oxidants is compromised then it leads to oxidative stress. In response to this stress, an inflammatory response is directed which results in the release of different cytokines and pro-inflammatory chemokines. This leads to the invasion of leukocytes and monocytes into the inflammatory environment. Continuous inhalation of injurious agents directs the initiation of prolonged oxidative stress in the lungs, which results in chronic inflammation characterized by a pronounced release of reactive nitrogen and oxygen species. This condition is related to significant DNA damage and genomic instability, leading to neoplastic progression and initiation (Valavanidis et al., 2013).

##### 3.1.1.6 Liver cancer

Different evidence has shown that oxidative stress plays a potent role in the development of hepatic carcinogenesis by disrupting genetic material, the normal functioning of the cell, and by interfering with various pathways of cell signaling (Jain et al., 2020). The application of antioxidant agents can limit oxidative stress-directed damages *in vitro*. But no drug is found to be effective under *in vivo* conditions with a smaller number of side effects. To find a more effective method for the cure and treatment of HCC, research for a better understanding of the mechanism involved in the development of cancer, OS-induced damage, and the effects of antioxidants is urgently required (Wang et al., 2016).

The liver is the main organ that is targeted by reactive species. Primary cells, i.e., parenchymal cells subjected to oxidative stress-directed damage in the liver (Jain et al., 2020). The peroxisomes, microsomes, and mitochondria in parenchymal cells lead the production of ROS by regulating the PPARα, which is involved in the expression of genes related to fatty acid oxidation. Moreover, endothelial cells, hepatic stellate cells, and kupffer cells are more sensitive towards oxidative stress-associated molecules. Different types of cytokines such as TNF-α can be released in Kupffer cells in response to oxidative stress, which might trigger the process of apoptosis and inflammation (Li S et al., 2015).

##### 3.1.1.7 Colorectal cancer

Colorectal cancer is the most common cancer globally, with the greatest incidence in Western nations. This cancer arises from the epithelial cells that line the bowel. The epithelial cells reported to have a great metabolic rate and divide rapidly which has been considered to be a potent factor responsible for the oxidation of DNA. Studies on rat colonocytes reported that the lower crypt cells are very much more sensitive towards hydrogen peroxide-induced damage than the differentiated cells located at the crypt surface (Perše, 2013). Since proliferating cells in the colon are present in the lower part of the crypt, this may determine that these cells are supposed to be the main targeted cells in case of colon carcinogenesis Progenitors or stem cells are very sensitive towards the redox environment. The differentiation and self-renewal of these cells are based majorly on the redox environment of the gut mucosa. Proliferating cells are also found to be sensitive to DNA damage as DNA is available in the form of a sgle strands in the S-phase of the cell cycle and further serves as a template in daughter cells. The damage of DNA in a single strand can lead to multiple mutations in the DNA of daughter cells, which could not get repaired (Oberreuther-Moschner et al., 2005). DNA damage causes genomic instability, replication error, induction of signal transduction pathways, induction of transcription and cell cycle arrest, all of which are related to colon carcinoma. However, the latest investigation has reported that the production of ROS may play a vital role in all the phases of carcinogenesis, i.e., progression, promotion, and initiation (Valko et al., 2007).

##### 3.1.1.8 Breast cancer

Breast tumor with increased proliferative capacity produces a great level of ROS during the chronic cycles of angiogenesis, reperfusion, and ischemia. Tumor cells can direct oxidative damage in surrounding healthy tissues. Enhanced levels of circulating malondialdehyde has been observed in the advanced stages of breast cancer as compared to early stages, which shows differences in the stage of oxidative stress. Patients with breast cancer have been reported to have a greater level of malondialdehyde, a marker of oxidative stress and peroxidation product, than the control patients (Coughlin, 2018). A breast tumor is a highly complex structure composed of old stromal and neoplastic cells. Carcinoma-associated fibroblasts (CAFs) has been usually reported in the cancer stroma, where they enhance vascularity and tumor growth. Under the influence of oxidative stress, fibroblast gets activated to become myofibroblast. These cells are greatly contractile and mobile and more often express mesenchymal markers. The activation of CAF is irreversible, which makes it difficult to be removed by nemesis. In cases of breast tumor, around 80% of stromal fibroblast needs an activated phenotype that manifests by the release of an enhanced level of metalloproteinase, cytokines, and growth factors. They also generate hydrogen peroxide, which directs the production of different sets of tumorigenic alterations and activated fibroblasts in the case of epithelial cells. Under oxidative stress, the tumor stroma produces a wide range of nutrients that support cancer cell survival and growth (Jezierska-Drutel et al., 2013).

##### 3.1.1.9 Prostate cancer

Prostate cancer is very common among males in Western nations. This chronic disease is very difficult to diagnose and has very limited treatment options. *In vivo* and *in vitro* studies determine oxidative stress as the main factor responsible for the occurrence of chronic prostatitis, prostatic cancer, and benign prostatic hyperplasia. Thus, the cascade of oxidative stress is the potential target for the cure of prostate-related disorders (Roumeguére et al., 2017).

#### 3.1.2 Roles of ROS in the pathophysiology of other diseases

##### 3.1.2.1 Liver diseases

The liver is the second largest organ in the organism’s body. It includes a broad range of vital functions such as storage of material absorbed from the digestive tract, hormone catabolism, synthesis of different compounds like clotting factors, globulin, and albumin and detoxification reactions. In alcoholic individuals, the main organ responsible for the removal of excessive alcohol is the liver and hence alcoholics are more prone to liver damage. Alcohol gets metabolized in the liver and during its metabolism, it generates free radicals. Metabolism of ethanol results in oxidative stress in hepatocytes through the generation of ROS. During metabolism, the molecules of alcohol break down into smaller molecules, which after further reactions leads to the production of ROS in the liver organ (Ceni et al., 2014), Figure 3. Alcohol promotes the production of ROS in various ways: 1) stimulating cytochrome P450s activity, 2) altering certain metal concentrations, 3) decreasing the antioxidants level, 4) directing the conversion of xanthine dehydrogenase (XDO) into xanthine oxidase (XO). These radicals raise peroxidation of lipids which leads to liver disorders such us: 1) Ischemic reperfusion injury (IRI): ROS generated by activated liver cells act as a vital mediator in liver ischemic-reperfusion injury (Zhang W et al., 2007) 2) Alcohol-induced oxidative stress: In ethanol-associated liver disorders, ROS plays an important role (Tan et al., 2020).

**FIGURE 3:**
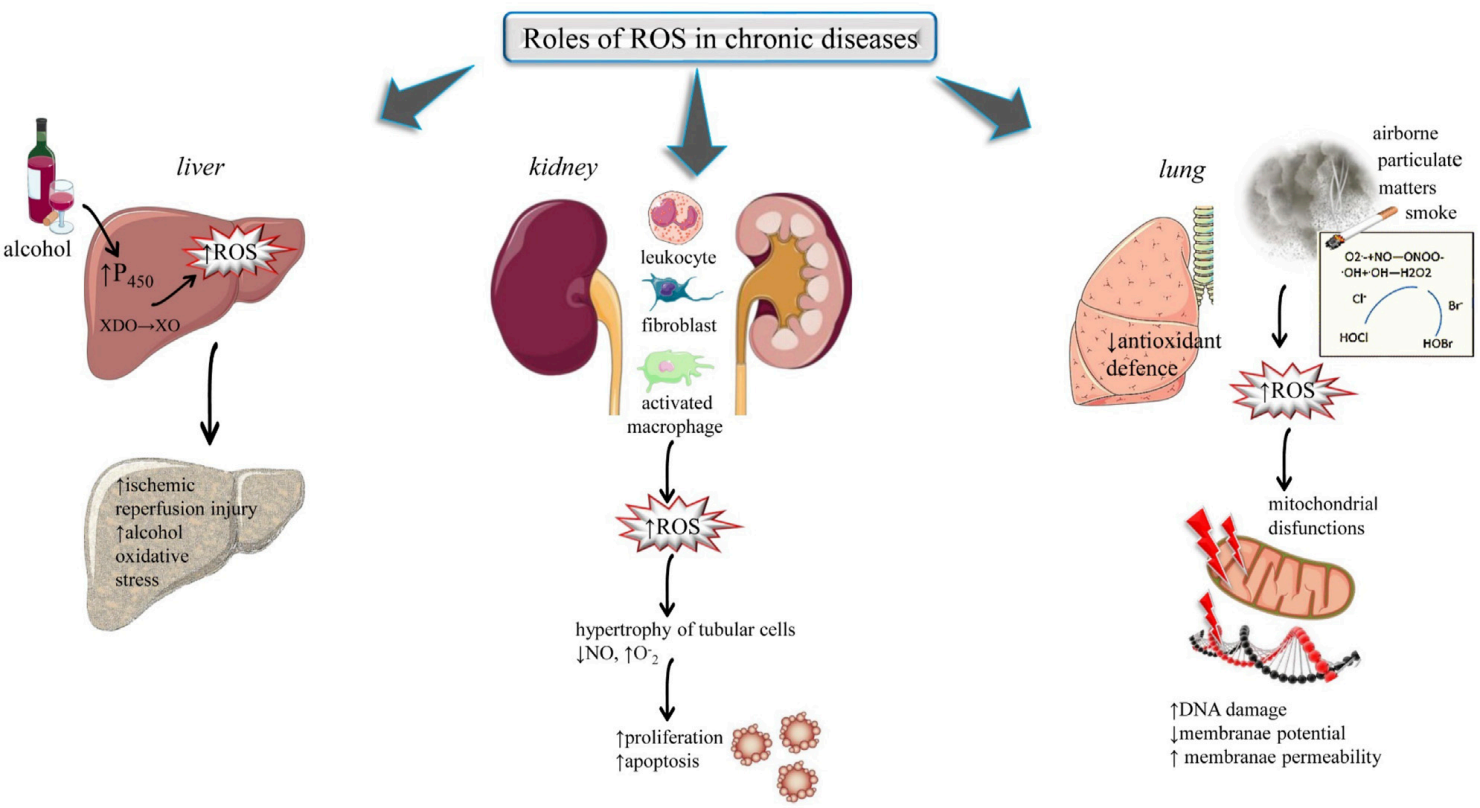
Highlights the impact of ROS in chronic diseases: liver, renal and kidney. Abbreviations and symbols: ↑ (increased), ↓ (decreased), ROS (reactive oxidative species), XDO (xanthine dehydrogenase), XO (xanthine oxidase), DNA (Deoxyribonucleic Acid).

##### 3.1.2.2 Renal diseases

The kidney is greatly vulnerable to alterations induced by ROS because of polyunsaturated fatty acids in the lipids of the kidney (Ander et al., 2003). The renal sources of ROS are leucocytes, fibroblasts, glomerular cells, interstitial cells, vascular cells, and activated macrophages. Oxidative stress played a potent role in various renal diseases such as tubule-interstitial fibrosis, ischemia-reperfusion injury, progressive and acute renal failure, and glomerulosclerosis (Ratliff et al., 2016; Duni et al., 2019), (Figure 3). Oxygen radical results in the following changes: 1) These free radicals result in hypertrophy of tubular cells, 2) Oxygen radical reacts with nitric oxide which is an endothelial vasodilator and inhibits its function. The products generated in this reaction are peroxynitrite (ONOO^−^) which results from the formation of hydroxyl radical (·OH), which is greatly reactive and results in the alteration of cellular functioning and cause dysfunction of endothelial cells, and 3) Massive generation of O_2_^−^ may result in proliferation and apoptotic death of epithelial cells (Pacher et al., 2007; Ratliff et al., 2016). Generally, renal patients required regular dialysis and this process undergoes the removal of small antioxidant molecules from the blood. This decreases the protection against reactive species and causes peroxidation of several biomolecules such as lipids. This raised the level of oxidative stress that can further lead to renal complications (Liakopoulos et al., 2017).

##### 3.1.2.3 Lung diseases

The lung organ exists in an oxygen-rich environment. Their large blood supply and greater surface area make this organ more susceptible to damage caused by oxidative stress. Exposure to exogenous ROS such as cigarette smoke, airborne particulate matter, and carbonyls/aldehydes, can result in oxidative stress and triggers the inflammation response in the lungs. On another side, an insufficient antioxidant defence mechanism in inflammatory cells, macrophages and lung epithelial cells can also result in high-level production of endogenous ROS in the lung tissues (Park et al., 2009; Pereira and Martel, 2014; Maynard, 2015; Bhatia, 2017). Mitochondria are majorly included in ROS-dependent pathways. Mitochondrial dysfunction also plays an important role in non-energetics pathogenesis and bioenergetics metabolism in different cases of lung disease. The genome of mitochondria acts as a guard to govern the cytotoxic response of pulmonary cells to oxidant stress (Schumacker et al., 2014; Kim et al., 2015). Mitochondria are included in ROS-initiated lung diseases such as lung cancer, chronic airway disease, asbestos, and lung fibrosis. The mitochondrial genome is more sensitive than the nuclear genome. Damage to mitochondrial DNA results in loss of mitochondrial membrane potential, impairment in the electron transport chain, and drives the immune and inflammatory responses (Liu and Chen, 2017), (Figure 3).

##### 3.1.2.4 Neurological disease

Neurodegenerative diseases are a heterogenous group of disorders that are characterized by the extensive loss of neurons. In the case of cerebral ischemia (CI), oxidative stress takes part in neuroinflammatory reactions. In postischemia brains, oxidative stress directs the activation of astrocytes and microglia which brings striking elevation in the inflammatory mediators such as matrix metalloproteases, chemokines, and cytokines and results in the loss of endothelial cell integrity in the brain by upregulating the neutrophil infiltration and cell adhesion molecules. M any studies reported that both nondopaminergic and dopaminergic cells undergo degeneration in the case of Parkinson’s disease. (Kim et al., 2015).

The innate mechanism of PD involves inflammatory responses and a spectrum of oxidative stress that results in neurodegeneration. Loss of dopaminergic neurons involves neuroinflammatory mechanism and oxidative stress through the elevated level of inducible nitric oxide synthase (iNOS) followed by activated astrogliosis, T-cell infiltration, and microglia that leads to the accumulation of NO and O_2_^−^ free radicals. Overexpression of cyclooxygenase-2 (COX2) is also responsible for dopaminergic neuronal loss *via* oxidative stress-mediated inflammation. In addition, the increasing level of myeloperoxidase by reactive astrocytes could also raise the level of reactive NO_2_^−^ and ∙OH radicals that could result in neuronal loss in Parkinson’s disease. Enhanced OS may direct mitochondrial dysfunction, impairment in the DNA repair system, and cellular damage, all of these processes have been regarded as the key factor responsible in the acceleration of the aging process and the initiation of other neurological diseases (Kim et al., 2015).

##### 3.1.2.5 Cardiovascular disease

ROS work as a secondary messenger within the heart as they are indulged in various physiological processes including contraction-excitation, proliferation, and differentiation. However, when the generation of ROS exceeds the level of antioxidant molecules in the heart, oxidative stress arises, which results from heart failure, cell death, hypertrophy, ischemia-reperfusion injury, and cardiac dysfunction. Endogenous ROSs in the heart are produced by uncoupled nitric oxide synthase, monoamine oxidases, cytochrome P450, NADPH oxidase, and xanthine oxidoreductase. ROS are also responsible for the initiation of some problems associated with specific clinical settings, comprising POAF, and chemotherapy-directed cardiotoxicity, as well as in the condition of diabetic cardiomyopathy, which determines a type of heart disorder in diabetic patients in the absence of other complications related to diabetes (D’Oria et al., 2020).

##### 3.1.2.6 Rheumatoid arthritis

Rheumatoid arthritis (RA) is a condition which gives rise to oxidative stress. A 5-fold increase in the concentration of mitochondrial ROS in monocyte and whole blood of RA patients is reported as compared with a healthy individual which suggests that OS has a pathogenic effect in RA. (Ponist et al., 2019; Yadav et al., 2023). Free radicals or reactive species are indirectly associated with joint damage, as they play the role of secondary messenger in the immune and inflammatory response in RA. The exposure of T-cells to raised OS become refractory to different stimuli including those for death and growth and may preserve the abnormal immune response. On the other hand, free radicals directly degrade the joint cartilage by targeting its proteoglycan and restricting its synthesis. Oxidative products of lipid peroxidation, oxidative damage of hyaluronic acid, and oxidation of low-density carbonyl and lipoproteins increment have been observed in RA patients. Raised levels of synovial fluid and 4-HNE have also been determined in the serum of RA patients (Ponist et al., 2019; Yadav et al., 2023).

##### 3.1.2.7 Cataract

A cataract is a partial or complete opacification on or in the human lens or in the capsule, which degrades vision. It is the main cause of reversible blindness in the globe today. It is determined that oxidation is an initial stage in the sequence of steps that lead to cataracts. Researchers have proposed a variety of factors which are related to the onset of cataractogenesis: enhanced permeability of the lens membrane, a decreased function of chaperone related to alpha crystalline, augmented non-enzymatic glycosylation, enhanced lipid peroxidation, and low capacity of antioxidant defence mechanism. These results have shown that the OS plays a major role in the pathogenesis of cataracts, which can be ameliorated and prevented by antioxidant molecules (Kaur et al., 2012).

## 4 Antioxidants: A brief synopsis

**FIGURE 4:**
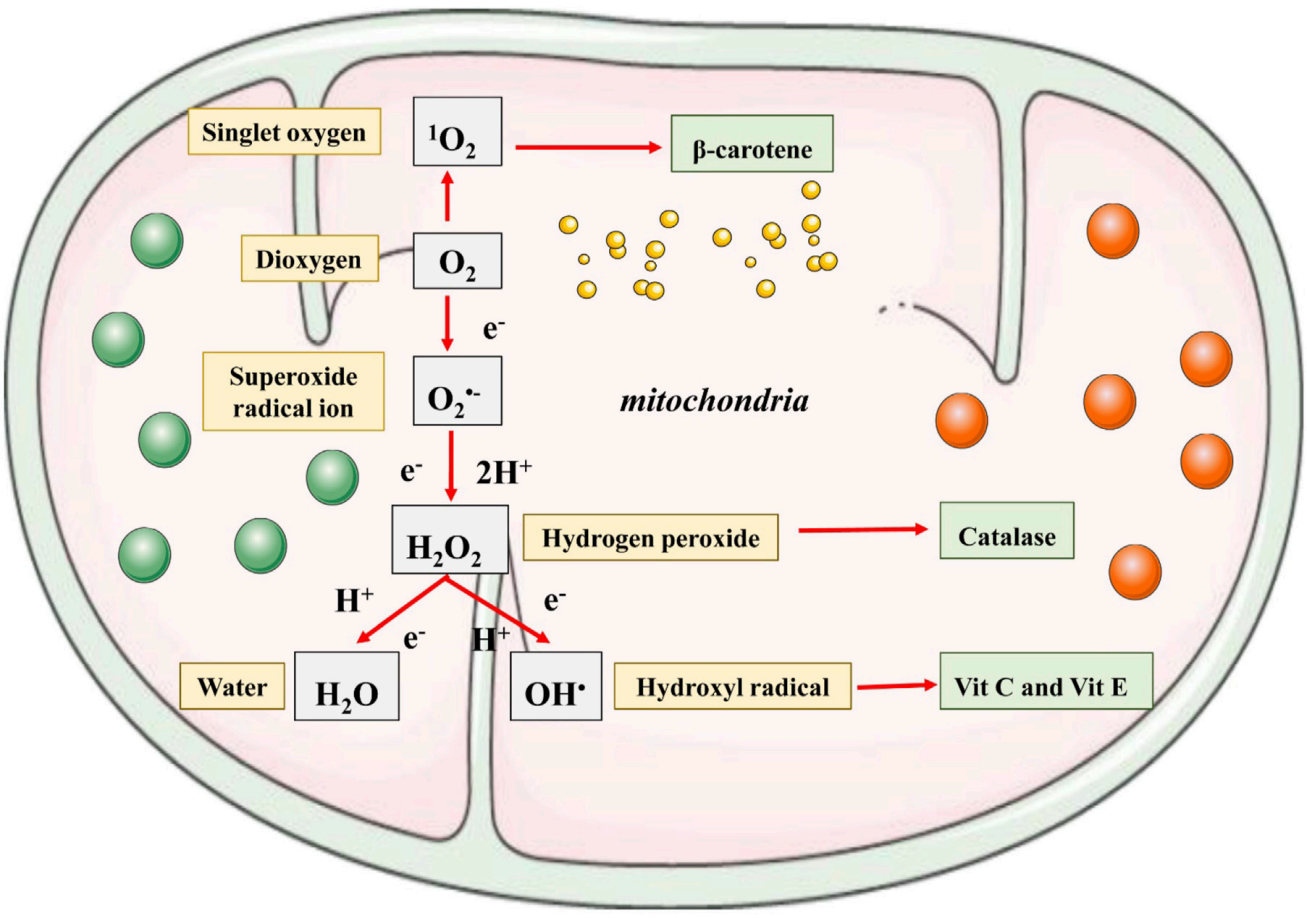
Summarized scheme regarding ROS generation at the mitochondrial level.

Mizanur Rahaman et al. (2023) Antioxidants work by preventing or delaying the oxidation of chemicals. The initial study on the antioxidant is majorly focused on their application in preventing unsaturated fats from getting rancid. However, the identification of vitamin E, C, and A in the living organism has led to an understandiof ng the function of antioxidants. They are usually divided into non-enzymatic and enzymatic. Among them, there are different types of compounds with different places amodesode of action and varied final effects (Flieger et al., 2021). They act as metal-chelatisynergists synergist, enzyme inhibitor, singscavengersn scavengedecomposerse decomposerdonorsctron donors, hydrogen donor, and radical scavenger (Sharifi-Rad et al., 2022). Both non-enzymatic and enzymatic antioxidants exist in extracellular and intracellular environment to detoxify the ROS. Two main principal mechanisms of action of that described for antioxidants are: The first mechanism is chain breaking in which primary antioxidants donated an electron to the free radicals whereas the second mechanism directs the removal of reactive species initiators (secondary antioxidants) by quenching the catalyst that initiate the chain of reaction. So, antioxidants extra t efonct in biological ssystemswith the help of various mechanisms such as gene expression regulation, co-antioxidants, metal ion chelation, and electron donation (Lobo et al., 2010). Antioxidants are the agent that when available in low concentration in comparison to the oxidizable substrate remarkably reduces or delays the oxidation of the substrate. The organism’s body has developed a different endogenous system to counteract the generation of reactive oxygen intermediates. The endogenous system is further categorized into non-enzymatic and enzymatic groups. The main function of antioxidants is to detoxify the reactive species in the body (Kurutas, 2016). Figure 4 shows the sites of action that are targeted by different antioxidants.

### 4.1 Role of endogenous/enzymatic antioxidants

Fortunately, the organism’s body contains a free radical defense mechanism. Every cell releases an antioxidant enzyme which protects the cells during the metabolism of oxygen, and further breaks down the harmful free radicals into balanced molecules like water (Lobo et al., 2010), (Table 2).

#### 4.1.1 Superoxide dismutase (SOD)

Living organisms have evolved a system for the scavenging of free radicals and reactive species. Superoxide dismutase carried out the removal of O_2_^•-^.

$${2O}_{2}^{•–}+{2H}^{+}\underset{SOD}{\to }\;H_{2}O_{2}+O_{2}$$

In another step, the hydrogen peroxide generated is eliminated by GPx and catalase system. Various isoenzymes of SOD are indulged in the scavenging activity of free radicals. The first isoenzyme is present in the mitochondria and is Mn^2+^ dependent whereas another isoenzyme is available in the cytoplasm and is dependent on Cu. Another extracellular Cu-Zn-dependent isoenzyme is also reported. Two different mechanisms have been reported for the SOD action that restricts the transformation 1) It majorly acts at the cell membrane level and eliminate or prevent the generation of radical that can result from the peroxidation of lipid and leads to the chain of extra-nuclear and nuclear events, ultimately cause transformation. This concept explains the model of a membrane that mediates chromosomal aberration, 2) It has been reported that oxygen radicals could be generated in a growth medium and act as a promoter of transformation that will be removed by SOD (Kowald et al., 2006).

#### 4.1.2 Catalase (CAT)

Catalase is present exclusively in peroxisomes. The purified form of catalase contains four subunits of protein, each of which has a heme (Fe III-protoporphyrin) group attached to its active site. Catalase is reported to play a dual role (Nandi et al., 2019).

1. A catalytic role in the decomposition of H_2_O_2_

$${2H}_{2}O_{2}\;\underset{Cat}{\to }\;{2H}_{2}O+O_{2}$$

2. A peroxidic role to oxidize a range of H donors (AH_2_)

$${AH}_{2}+H_{2}O_{2}\;\underset{Per}{\to }\;A+{2H}_{2}O$$

DNA cleavage by X-ray and mitomycin C induced malignant transformation which was found to be suppressed by the catalase enzyme. According to some studies, factors such as brain-derived neurotrophic factor (BNDF) and stress are responsible for the antioxidant activity of several endogenous antioxidants (Lobo et al., 2010). The mice with BNDF deficiency under the stress circumstances showed increased activity of catalase enzyme in comparison to the stressed wild type. This indicates that the capacity to scavenge free radicals was reduced and this determines that the normal wild type has better tolerance capability than the BNDF heterogeneous mice (Hacioglu et al., 2016).

#### 4.1.3 Glutathione systems

The system of glutathione includes glutathione-S-transferase, glutathione peroxidase, glutathione reductase, and glutathione. This system is reported in microorganisms, plants and animals. Glutathione peroxidase is an enzyme that contains 4 units of selenium as cofactors which catalyzes the breakdown of organic hydroperoxides and hydrogen peroxides. About four different isoenzymes of glutathione peroxidase are reported in animals. Glutathione peroxidase 1 is the efficient scavenger of hydrogen peroxide while glutathione peroxidase 4 is found to be more effective with lipid hydroperoxides. The glutathione S-transferase shows greater activity with lipid peroxides. These enzymes are present at high concentrations in the liver and also help in the metabolism of detoxification (Lobo et al., 2010).

Glutathione is a non-protein thiol that coordinates the processes of antioxidant defense in the body. Alterations in the status of glutathione have been reported to cause several complications. Administration of thiol compounds such as methionine, cysteine, and glutathione are known to protect against oxidative stress in animals and humans. The reduced form of thiols has been utilized for the recycling of other antioxidants such as vitamin C and vitamin E (Noctor et al., 2011).

#### 4.1.4 Glucose-6-phosphate dehydrogenase

Protection against OS is largely depending upon the reductive power of NADPH, whose level is evaluated with the help of glucose-6-phosphate dehydrogenase (G6PD). Animal cells contain few enzymes that can generate NADPH, and among them, G6PD is the most important one (Peters and Van Noorden, 2009). G6PD catalysis is the rate-limiting step of the pentose phosphate pathway (PPP), which provide nucleotide precursors for DNA replication, as well as reductive power to NADPH for the detoxification of ROS and *de novo* synthesis of lipid (Stanton, 2012). The relation of PPP and G6PD in the detoxification of ROS is determined by the fact that mice deficient in G6PD have higher levels of oxidative damage in the brain. Animal cells also respond against oxidative stress by enhancing their PPP-directed NADPH production. The overexpression of G6PD in *Drosophila melanogaster* protects against oxidative stress and can also expand their lifespan (Nóbrega-Pereira et al., 2016).

### 4.2 Role of exogenous antioxidants

#### 4.2.1 Vitamins and selenium

Exogenous antioxidants like vitamins E, C, and A played a supporting role. They scavenge the free radicals and reactive species by donating an electron and maintaining a chemical balance. These dietary agents get saturated easily, only once as they donate the electron (Table 1).

**TABLE 1:** Classification of major antioxidants and their roles.—this Table must totally re-written/updated according to the reviewer 3 comments.

	Antioxidant	Role	References
Enzymatic	Superoxide dismutase (SOD)	Dismutates O_2_^•⁻^ to O_2_ with a higher rate of reaction by successive reduction and oxidation of transition metals to H_2_O_2_	Buettner (2011)
	Catalase	Dismutates H_2_O_2_ to H_2_O and oxygen	Nandi et al. (2019)
	Glutathione peroxidase (GPx)	↓lipid peroxides, ↓H_2_O_2_ in the presence of tripeptide glutathione, GPx added two electrons to carried out the reduction of peroxides	Lubos et al. (2011)
Non-enzymatic (Vitamins)	Alpha-tocopherol	major membrane bound antioxidant	Traber and Stevens (2011)
		↓lipid peroxide scavenge O_2_^•, •^OH radicals	
		interrupt the chain reaction of free radicals by capturing free radicals	
	Beta carotene	scavenges peroxy, O_2_^•**⁻**^ **,** ^**•**^OH radicals	Grune et al. (2010)
		↓vitamin A oxidation	
	Ascorbic acid	↓ H_2_O_2,_ ↓^•^OH, ↓ O_2_^•**⁻**^ radicals; neutralizes oxidants obtained from stimulated neutrophils	Traber and Stevens (2011)
		↑ vitamin E	
Non-enzymatic	Carotenoids	restrict the propagation of free radical chain reaction	Jomova and Valko (2013)
Non-enzymatic	Flavonoids	stabilizes the ROS	Panche et al. (2016)
Non-enzymatic	Melatonin	detoxify reactive nitrogen and oxygen species and raises the activity of the antioxidant defense system	Hacişevki and Baba (2018)
Non-enzymatic	Thiol antioxidants (lipoic acid, thioredoxin, and glutathione)	thiol, compounds showed antioxidant activity due to the presence of sulphur atoms	Flora (2009)

##### 4.2.1.1 Vitamin E

Vitamin E (alpha-tocopherol) is considered to be an important antioxidant that involves in the chain-breaking process in the case of humans. Vitamin E is present within the cell membrane to interrupt the peroxidation of lipid and also play a major role in the modulation of cell signaling pathways that are dependent on reactive oxygen intermediates (ROI). They also can directly scavenge the reactive species, including ^•^OH, O_2_^•-^, and ^1^O_2_ (Traber and Atkinson, 2007). Vitamin E also plays a potent role in decreasing the cases of cancer. It functions by donating the hydrogen atom to fatty peroxyl radicals and disrupting the lipid peroxidation process. It also has the potential to make a reaction with two peroxyl radicals as shown below:

$$\alpha -tocopherol+{LOO}^{-}\;\underset{Vit\;E}{\to }{\alpha -tocopherol}^{.}+LOOH$$

α−tocopherol•+LOO• LOO–α−tocopherol

$$\alpha -tocopherolthe+{LOO}^{.}\;\underset{Vit\;E}{\to }\;LOO-\alpha -tocopherol$$

The vitamin E synthesized in Equation assumes to have a resonance-stabilized conformation which allows this vitamin to react with other peroxyl radicals to form a stable adduct, LOO-α-tocopherol. Alpha-tocopherol restricts the generation of new free radical whereas gamma-tocopherol neutralizes or trap the existing reactive species. OS has been associated with various possible diseases but rather vitamin E helps to delay or prevent the chronic ailments associated with free reactive molecules (Traber and Stevens, 2011).

##### 4.2.1.2 Vitamin C

Uric acid, cysteine, glutathione, and vitamin C (ascorbic acid) work as a hydrophilic scavenger of oxygen radicals (Lobo et al., 2010). Natural ascorbate restricts the carcinogenic process of various nitroso compounds, fed to animals by converting them to an inactive form. This vitamin C can be utilized to detoxify the different organic compounds *in vivo* by a simple reduction process (Combet et al., 2007). Ascorbic acid is a potent scavenger and reducing agent of reactive species in the biological system. It is included in the first line of ddefenceof antioxidants, protecting the proteins and lipid membrane from damage (Pehlivan, 2017). Due to its water-soluble nature, vitamin C can work both outside and inside the cells and can neutralize the free reactive species to avoid any damage. This vitamin works as an excellent source of electrons for the reactive species that are finding out an electron to acquire their stability. They can donate electrons to those free radicals and scavenge their reactivity (Padayatty and Levine, 2016). Cysteine is the physiological precursor of glutathione (GSH) and has been known widely for its protective function against mutagenesis and radiation. Vitamin C or ascorbic acid reacts with singlet oxygen (^1^O_2_), peroxyl radical (ROO•), hydrogen peroxide (H_2_O_2_), and hydroxyl radical (OH), to form the dehydroascorbate A) and semidehydroascorbate radical (A‾) as shown in equation below (Phaniendra et al., 2015).

$${AH}^{-}+•OH\;\underset{Vit\;C}{\to }\;H_{2}O+A^{•‾}$$

$${AH}^{‾}+{1O}_{2}+H^{+}\;\underset{Vit\;C}{\to }\;H_{2}O_{2}+A^{•-}$$

$${AH}^{‾}+ROO•\;\underset{Vit\;C}{\to }\;RH+A^{•‾}$$

$${AH}^{‾}+H_{2}O_{2}+H^{+}\underset{Vit\;C}{\to }\;{2H}_{2}O+A$$

##### 4.2.1.3 Vitamin A

Vitamin A and *ß*-carotene are important antioxidants as they interfere with the process of peroxidation. *ß*-Carotene is also found to be useful in decreasing the incidence of cancer. Two major functions of *ß*-carotene are the ability to neutralize and trap certain organic free radicals and to deactivate the oxygen radicals present in exciting form, produced as a byproduct of metabolic reactions (Fiedor and Burda, 2014). The consistent and strongest conformation regarding the protective effect of large consumption of carotene-rich food comes from studies of the esophagus and lung cancer (Patel et al., 2018). *ß*-Carotene act as an antioxidant under low oxygen tension and can also act as a prooxidant under more oxidizing and high concentration level in the case of smokers (Tapiero et al., 2004).

The reaction of *ß*-carotene (CAR) with lipid peroxyl radicals (LOO•) to form various carbon-centred radicals such as (LOO)_2_-CAR-(OOL)_2_) (LOO)_2_-CAR-OOL•, LOO-CAR-OOL, and LOO-CAR• is represented in equation.

$$CAR+{LOO}^{•}\;\underset{\beta -caro}{\to }\;{LOO-CAR}^{•}$$

$${LOO-CAR}^{•}+LOO\;\underset{\beta -caro}{\to }\;LOO-CAR-OOL$$

$$LOO-CAR-OOL+{LOO}_{2}^{•}\underset{\beta -caro}{\to }\;{\left(LOO\right)}_{2}{-CAR-OOL}^{•}$$

$${\left(LOO\right)}_{2}{-CAR-OOL}^{•}+{LOO}^{•}\underset{\beta -caro}{\to }\;{\left(LOO\right)}_{2}{-CAR-\left(OOL\right)}_{2}$$

A sole molecule of *ß*-carotene is determined to eliminate around 1,000 of singlet oxygen with the help of a physical mechanism which involves the transfer of energy before it gets oxidized and loses its antioxidant activity (Tan et al., 2018). The rate of *ß*-carotene oxidation is dependent upon the partial pressure of oxygen. The carbon-centred radicals get resonance stabilized when the oxygen pressure is lowered. The balanced reaction rapidly shifts to the left side to lower the concentration of peroxyl radicals (LOO•), and the autooxidation rate of *ß*-carotene get reduced (Iannone et al., 1998). The *ß*-carotene activity towards peroxyl radicals and the stability of other carbon-centred radicals are the two main features that provide the carotene molecule with its antioxidant properties (Fiedor and Burda, 2014).

##### 4.2.1.4 Selenium

Selenium is a natural element which has gained focus due to its potential to decrease the process of carcinogenesis. The antioxidant activity of selenium concerning vitamin C was studied broadly (Tan et al., 2018). Deficiency of selenium results in the consequent cellular and tissue damage raised peroxidation of lipids and leads to the formation of free radicals. Damage caused to unsaturated fatty acids in the subcellular membrane by peroxidation reaction can be reduced by Se and vitamin E (Mourente et al., 2007). The metabolisms of Se and vitamin E are interrelated and Se plays a major role in vitamin E storage.

#### 4.2.2 Natural antioxidants

##### 4.2.2.1 Phenolic compounds

Phenolic compounds are a heterogenous group of phytocompounds that are broadly spread in the plant kingdom (Chaudhary et al., 2023). Flavonoid belongs to a class of naturally occurring polyphenolic compounds and they provide different colors to leaves, fruit, and flower (Chaudhary et al., 2023). Flavonoids are further divided into the following classes: anthocyanidins, flavones, flavonols, flavanones, and flavonols. Among the classes, variations are based on the arrangement and number of hydroxyl groups, and glycosylation or alkylation of these groups. The broad varieties of flavonoids and the greatest difference in their content make it difficult to determine the daily intake estimate of flavonoids (Panche et al., 2016). Phenolics and flavonoids act as an antioxidant through several pathways The most potent one is likely to be by scavenging the free radicals in which polyphenols carried out the breakdown of several free radical chain reactions (Kaurinovic and Vastag, 2019). For a molecule to act as an antioxidant, it must fulfil the two criteria.

(i) at low concentrations, it can prevent the oxidation of the substrate
(ii) the radical generated on the polyphenols must be stable enough so that it can prevent itself from acting as a chain-propagating radical (Dangles, 2012).

This stabilization is usually through intramolecular hydrogen bonding and delocalization or by reaction with other lipid radicals and further oxidation. A large number of investigations have been done on the structure-antioxidant activity relationship of flavonoids (Table 2). Some of the common ones are flavanol, flavonoid, and quercetin which are abundant in onion, broccoli, and apple; catechin, another flavanol present in different tea and fruits; naringenin, the major flavanone in grapefruit; glycitein, genistein, and daidzein are the major isoflavanones in soybean; and cyanogen glycosides are abundant in blackberry, raspberry, and black currant.

**TABLE 2:** The role of natural antioxidants in the chemoprevention of cancer.

Dietary agent	Chemopreventive effects	References
Soy (Flavonoids, Genistein)	control cell cycle, ↓topoisomerase IIantioxidant	Mehta et al. (2010)
Ursolic acid	regulate pro-and anti-apoptotic proteins	Iqbal et al. (2018)
	↓free radicals, ↑arrest cell cycle, ↑apoptosis	
	↓angiogenesis, ↓proliferation, ↓metastasis	
β-caryophyllene	analgesic	Fidyt et al. (2016)
β-caryophyllene oxide	anticancer properties	
Capsaicin	↑autophagy, ↑apoptosis, ↓angiogenesis	Zhang J. et al. (2019)
	↓proliferation, ↓metastasis	
Deoxyelephantopin Isodeoxyelephantopin	↑ apoptosis	Mehmood et al. (2017)
Zingerone	↑apoptosis	Su et al. (2019)
Isoliquiritigenin	↓angiogenesis	Wang et al. (2013)
Indole-3-carbinolGenistein	↑ apoptosis	Ushio-Fukai and Nakamura (2008)
Epigallocatechin gallate	↑apoptosis, ↓cell growth, ↓NF-kB	Khan et al. (2010)
Sulforaphane	↓histone deacetylase	Ho et al. (2009)
Chrysin	chemopreventive effect against benzo(a) pyrene-induced lung cancer in swiss albino mice	Kasala et al. (2016)
Mangiferin	↓cell-cycle proteins	Rajendran et al. (2015)
	↓cytokines, ↓inflammatory markers	
Piperlongumine	↓NF-kB, ↓ metastasis, ↓cancer cells growth	Kumar and Agnihotri (2021)
6-shogaol	↑anticancer effect of irinotecan, oxaliplatin, 5-fluorouracil	Woźniak et al. (2020)
	↑autophagy, ↑apoptosis on colon cancer cells under hypoxic conditions	
Allicin	↓invasion, ↓ proliferation of cells by ↓STAT3 signaling in the case of cholangiocarcinoma	Chen et al. (2018)
Baicalein	↓invasion, ↓proliferation of cells, ↓snail-induced epithelial-mesenchymal transition in case of colorectal cancer	Zeng et al. (2020)
Gingerol	↑apoptosis, ↑caspases, ↓proliferation of cancer cells	Radhakrishnan et al. (2014)
	↓ MAPK/AP-1 signaling in case of colon cancer cells	
Glycyrrhizic acid	↓proliferation of cells, ↑apoptosis, ↑cell cycle arrest in the case of gastric cancer cells	Wang H et al. (2020)
Hispidulin	↑anticancer activity, ↑endoplasmic reticulum stress in the case of non-small cell lung cancer	Lv et al. (2020)
Licochalcone A	↓proliferation of cells, ↑ATM-CHEK2, ↑autophagy	Shen et al. (2019)
Gallic acid	↓NF-kB	Morais et al. (2010)
Curcumin	↓CDK-4 a, ↓Cyclin D1 in the case of breast cancer cells	George et al. (2021)
Genistein	↓cancer cell proliferation	
	↓PTK signaling, ↓protein-tyrosine kinase	
Apigenin	↓ HPV-induced prostate cancer	
	↑apoptosis, alteration of cell cycle	
Synthetic agents	Chemopreventive effects	References
2′,5′-dialkoxylchalcones	↓tubulin polymerization	Cheng et al. (2008)
1,4-Phenylenebis (methylene)selenocynate	↓colon tumor incidence	Reddy and Lokesh (1992)
butylated hydroxytoluene	protective effect against ferric nitrilotriacetate induced oxidative stress and hepatotoxicity	Ansar et al. (2013)
Butylated hydroxyanisole	↓ARE-mediated expression of gene	Yuan et al. (2006)
	↓Nrf2 coupled with JNK	
	↓ERK signaling pathway in HepG2 cells	

Abbreviations and symbols:↑ increase, ↓decrease, NF-kB: Nuclear factor kappa B; STAT3: Signal transducer and activator of transcription 3; MAPK/AP-1: Mitogen-activated protein kinase-Activator protein-1; ATM-CHEK2: Ataxia telangiectasia mutated serine-checkpoint kinase 2; CDK-4: Cyclin-dependent kinase 4; PTK: protein tyrosine kinase; ERK: Extracellular signal-regulated kinase; HepG2: liver cancer cell line.

##### 4.2.2.2 Curcumin

Curcumins target free radicals through various mechanisms. It can restrict the activation of ROS-producing enzymes such as xanthine oxidase/hydrogenase, and cyclooxygenase/lipoxygenase, it can modulate the activity of SOD, catalase, and GSH enzymes for the neutralization reaction, and it can also have the ability to scavenge the reactive nitrogen and oxygen species. Additionally, curcumin is a lipophilic molecule, which makes it useful in the scavenging of peroxyl radicals. Therefore, like vitamin E, curcumin is also utilized as a chain-breaking natural agent.

##### 4.2.2.3 Tannins

Tannin is the common name for phenolic molecules that are used for tanning leather and the precipitation of gelatin from solution. They are further categorized into the condensed form of proanthocyanidins, in which the main structural unit is the phenolic flavan-3-ol nucleus and hexahydroxydiphenoyl and galloyl ester and their derivatives, ellagitannins and gallotannins (Clifford and Scalbert, 2000). The two main groups of phenolic acids are hydroxycinnamic acid and hydroxybenzoic acid, both of which are obtained from cinnamic acid and benzoid molecule, respectively (Kumar and Goel, 2019). These phenolic acids contain benzoic acid derivatives such as cinnamic acid derivatives (ferulic, caffeic and coumaric acid) and gallic acid. Caffeic acid is majorly found in vegetables and fruits, most commonly esterified with quinic acid as in chlorogenic acid, the main phenolic in coffee. Another common phenolic acid is ferulic acid which is esterified with hemicellulose and present in cereals (Dai and Mumper, 2010).

##### 4.2.2.4 Phenolic acids

###### 4.2.2.4.1 Caffeic acid

Caffeic acid is produced by different plant species and is found to be available in various food products such as tea, wine, and coffee and other medicines such as propolis. Phenolic acid and its derivatives have anticarcinogenic, anti-inflammatory, and antioxidant activities. *In vivo* and *in vitro* investigations have determined the anticarcinogenic property of this agent in the case of hepatocarcinoma, which is considered to be a highly aggressive form of cancer, responsible for the large rate of mortality across the globe. The anticancer activity of this compound is related to its pro-oxidant and antioxidant capacity due to its complex structure including double bond in the carbonic chain, the position and number of OH group and free phenolic hydroxyl in the catechol group, respectively (Kiokias et al., 2020). The pharmacokinetic investigation determined that this compound is being hydrolyzed by the microbial colonies and metabolized in the mucosa of the intestine through phase II enzymes, submitted to methylation and conjugation process, forming methylated, glucuronic, and sulphated conjugates by the action of o-methyltransferase, UDP-glucosyltransferases, and sulfotransferases, respectively (Monteiro Espindola et al., 2019). The transmembrane flux of this compound in intestinal cells occurs *via* the active transport carried out by the carriers of monocarboxylic acid. It can act by suppressing the expression of MMP-9 and MMP-2, blocking STATs, reducing the angiogenesis of tumor cells, inducing the oxidation of DNA of tumor cells, and preventing the generation of ROS (Monteiro Espindola et al., 2019).

###### 4.2.2.4.2 Gallic acid

Gallic acid exerts its antioxidant effects by modulating the pro-oxidant/anti-oxidant balance. It can induce apoptosis, autophagy, and cell cycle arrest *via* activating the ROS generation and caspase pathway. Additionally, they can restrict metastasis and invasion by reducing the activity and expression of matrix metalloproteinase (Kahkeshani et al., 2019).

###### 4.2.2.4.3 Ferulic acid

The antioxidant activity of ferulic acid is very complex. It is majorly based on the restriction of RNS and ROS formation and neutralization of free radicals. This compound also acts as a hydrogen donor; donating atoms directly to free radicals. This acid is found to be important for the protection of lipidic acids in the cell membrane from the unwanted autoxidation process. As a secondary compound, ferulic acid and its derivatives can bind with copper and iron and avoid the generation of toxic hydroxyl radicals, which direct cellular damage (Zduńska et al., 2018).

###### 4.2.2.4.4 Carnosic acid

This acid is extracted from *Rosmarinus* and *Salvia* species and reported with various antioxidant and functional properties. It is most commonly used in the pharmaceuticals and cosmetic sector. In *vitro* experimental investigation, it has been observed that the administration of 20 μg/mL of Car A resulted in the cure of breast cancer *via* the activation of apoptotic and antioxidant genes (Einbond et al., 2012).

###### 4.2.2.4.5 p-Coumaric acid

This acid is most commonly found in cereals, vegetables and fruits. It acts as a potent antioxidant and scavenges free radicals and reactive species. p-coumaric acid is a phenolic acid and is a hydroxyl derivative of cinnamic acid. It reduces the prefigure oxidation of low-density lipoprotein and decreases the risk of stomach cancer (Kiliç and Yeşiloğlu, 2013).

#### 4.2.2.5 Stilbenes

Stilbenes comprise two phenyl moieties which are connected by the methylene bridge of two carbon atoms. The availability of stilbenes in an organism’s diet is very low. Majorly these compounds act as an antifungal phytoalexin, the compound which is synthesized in response to injury. One of the widely known stilbenes is resveratrol, found majorly in grapes. Products of red wine and grapes contain a significant amount of resveratrol, respectively (Rocha-González et al., 2008).

#### 4.2.2.6 Lignans

Lignans are the phenolic compounds and have 2,3-dibenzylbutane structures that is resulted from the dimerization of two cinnamic acid residues; lignans such as secoisolariciresinol are considered phytoestrogen. The increasing interest in the beneficial effect of phenolic compounds has resulted in the development of such diets that are rich in vegetables and fruits and provide protection against cancer and cardiovascular diseases. The linseed act as a source which provides secoisolariciresinol (up to 3.7 g/kg dry weight) and a low amount of mataresinol, respectively (Milder et al., 2005).

#### 4.2.2.7 Alkaloids

*Erythroxylum cuneatum*, a tropical flowering plant of the Erythroxylaceae family utilized in Thailand and Malaysia as traditional medicine. The alkaloid extract of E. *cuneatum* leaf possesses both anti-inflammatory and antioxidative activity suggesting its role in the development of anti-inflammatory and antioxidant drugs (Li S et al., 2015). Two isoquinoline alkaloids kareemine 2) and iraqiine 1), along with N-methylouregidione 7), atherospermidine 6), O-methylmoschatoline 5), kinabaline 4), and muniranine 3) were isolated from the dichloromethane extract of *Alphonsea cylindrica* bark. Compounds 4, 3, and 1 possess higher DPPH scavenging activity (Obaid Aldulaimi et al., 2019). The antioxidant activity of alkaloid boldine and *Peumus boldus* extract was reported against Fe(II)-citrate-induced damage in rat liver mitochondria *in vitro* (Klimaczewski et al., 2014). *Stephania rotunda* Lour., a Cambodian wild plant utilized in traditional medicine and food for the treatment of fever. The antioxidant activity of fangchinoline and cepharanthine was reported from *Stephania rotunda* by *in vitro* assays (Gülçin et al., 2010). The results determined an effective radical scavenging and antioxidant activity of fangchinoline and cepharanthine. Five new alkaloids (1–5) along with two new phenanthrene and three aporphine alkaloids, in total 10 (6–15) compounds were isolated from the roots of *Stephania tetandra*. Based on electronic circular dichroism, single crystal X-ray, and spectroscopic analysis, compound 13, and 7–10 exhibited great antioxidant activities (Wang R et al., 2020).

#### 4.2.2.8 Terpenoids

Terpenes play an important role in the metabolic processes of a broad range of microorganisms, plants, and animals in which they are formed Naturally, terpenoids can be utilized for different purposes including as key agents in metabolic processes, signaling, and defense (Baccouri and Rajhi, 2021). These terpenes have had applications in medicine, cosmetics, and perfumery for thousands of years and are still procured from different plants for the above-mentioned uses. The antioxidant activities of terpenes may explain their capacity to adjust the neural signal transmission, immunological effects, and inflammation. They protect against oxidative stress situations including ageing, diabetes, neurodegenerative, cardiovascular disease, cancer, liver, and renal mechanisms (Baccouri and Rajhi, 2021). Bourgou et al. (2012) reported the antioxidant activity of isolated terpenoids from Tunisian *Nigella sativa* L. essential oils and also their ability in restricting the production of nitric oxides. The chemical characterization and antioxidant activity of essential oils isolated from *Euphorbia heterophylla* L. (Elshamy et al., 2019) and their allelopathic potential were reported against *Cenchrus echinatus* L. Sesquiterpenes-rich essential oil isolated from the above parts of *Pulicaria somalensis* showed great antioxidant activity and allelopathic effect against weeds (Assaeed et al., 2020). In a study to determine the antitumor effects of terpenoids, it was found that paclitaxel, geraniol, and perillyl alcohol are the terpenoids with better anticancer activities (Yang et al., 2020). The anti-inflammatory activity of paeoniflorin and its derivatives, 4-O-methylbenzoyl paeoniflorin, 4-O-methyl paeoniflorin, and other monoterpenes was reported (Bi et al., 2017). The results reported that most of these monoterpenes can inhibit the production of tumor necrosis factor-alpha (TNF-α), interleukins-6 (IL-6), and inflammatory factor nitric oxide (NO) induced by lipopolysaccharides (LPs).

## 5 Antioxidants: Mode of action and molecular mechanisms

The mode of action of antioxidants can be explained by the following routes.

### 5.1 Preventive antioxidants

ROS such as OH, O2•, and H_2_O_2_ are generated irreversibly during the process of metabolism. Other free radicals such as ROOH, and organic hydroperoxide are generated by the reaction of radicals with the cellular components such as nucleobases and lipids. The RO, ROO. peroxyl, and alkoxy radicals are oxygen centered organic types of radicals. Lipids form take part in the peroxidation reaction of lipids. They are generated in the presence of oxygen by abstraction of hydrogen and addition of radicals to double bonds. Hypochlorous acid or HOCl are generated from hydrogen peroxide in the presence of myeloperoxidase. They are highly reactive and readily oxidized protein constituents such as methionine, amino groups, and thiol groups. Peroxynitrite radical is generated in a reaction of superoxide with nitric oxide. The protonation reaction results in the formation of peroxynitrous acid which undergo hemolytic cleavage to form nitrogen dioxide and hydroxyl radicals (Lobo et al., 2010). Therefore, various methods have been applied to decrease the damage caused by oxidative stress. Endogenous antioxidant enzymes which are generated inside the cells work as a protective mechanism against reactive free species. Heme oxygenase, reductase, thioredoxin, GST, GR, GPx, CAT and SOD are the most vital antioxidant enzymes. The SOD carried out the conversion of O2^•–^ into H_2_O_2_, which is further converted into H_2_O with the help of the Fenton reaction, GPx, and CAT. Thus, there is a conversion of toxic species to the harmless product. Peroxides generated during the process of metabolism, get eliminated by GPx and GST. GRd is found to be useful to maintain the equivalent of oxidized glutathione (GSSG) and GSH, as the ratio of GSSG/GSH is an indicator of oxidative stress. So, GRd functions by raising the concentration of GSH, which is required for the maintenance of oxide-redox conditions in a living organism. The GPx is available throughout the cells whereas CAT is restricted to the peroxisome. The brain is very sensitive towards the damage caused by free radicals so; it contains 7 times more concentration of GPx than the CAT. The greatest level of CAT is found in erythrocytes, kidneys, and the liver, where it decomposes most of the hydrogen peroxide (Aziz et al., 2019).

### 5.2 Free radical scavengers

The utilization and consumption of oxygen in physiological processes result in the production of ROS. The production of energy in mitochondria is dependent upon the metabolism of oxygen since oxygen gets reduced to water. During the transfer of electrons through a pathway, incomplete reduction of oxygen can result in the production of highly damaging and reactive ROS, such as hydroxyl radical, hydrogen peroxide, singlet oxygen and superoxide radicals The toxicity of superoxide radicals can be diminished with the help of metalloenzyme SOD which catalyzes the reduction of O_2_^•-^ to O_2_ and H_2_O_2_. Other antioxidant agents such as green tea extract are also capable of reducing the damage resulting from superoxide radicals. Hydroxyl radicals are generally formed from the Cu^+^/H_2_O_2_ or Fe^2+^ Fenton reaction system *via* incubation of H_2_O_2_ and FeSO_4_ in an aqueous solution. These radicals are found to be toxic to macromolecules such as proteins, lipids, and DNA. The application of various polyphenol and polyene from different vegetables and fruits protect against the damaging effect of hydroxyl radicals (Lipinski, 2011). Large numbers of metals are responsible for inducing carcinogenicity and toxicity in the animal body. An excessive amount of iron in the body leads to cancer, vascular diseases and other neurological complications. Copper at higher concentrations is known to result in metastasis. The complexes of cobalt ion led to the production of ROS which cause heart complications. Research showed that Se can chelate copper ions efficiently and prevent the damage caused to DNA by hydroxyl radicals. The component of red wine binds to high-density lipoprotein (HDL) and low-density lipoprotein (LDL) and protect lipoproteins from metal-independent and meta-dependent lipid and protein oxidation (Ivanov et al., 2001). According to some studies, melanoidin possessed better scavenging and metal-ion chelating activity which were due to its molecular weight (Wu et al., 2021). It has been determined that the major source of free radicals in diverse pathological and physiological circumstances is related to the enzyme number. The enzymes that lead the generation of superoxide include NADPH-dependent oxidase, cyclooxygenase, lipoxygenase, and xanthine oxidase. The production of hydrogen peroxide is catalyzed by the superoxide dismutase enzyme. Various enzymes of peroxisomes such as D-aspartate oxidase, xanthine oxidase, urate oxidase, D-amino acid oxidase and acyl CoA oxidase direct the production of different ROS. Many natural agents have revealed their potential to restrict the enzymes that direct the generation of free radicals as well as in the development of novel therapeutics agents against oxidative stress-induced diseases (Phaniendra et al., 2015). Plant alkaloids such as berberine carried out the inhibition of NADPH oxidase activity *via* reducing the mRNA expression of gp91phox in macrophages. Similar results were obtained by the treatment with dihomo-*γ*-linolenic (*ω*-6) acid, ellagic acid (from nuts and fruits), and 3-(4′-hydroxyl-3′,5′-dimethoxyphenyl) propionic acid (HDMPPA) (from kimchi), (Maraldi, 2013). Eugenol obtained from *Ocimum sanctum* showed 97% inhibition in the activity of cyclooxygenase at 1000-microM concentration (Kelm et al., 2000).

### 5.3 Prevention of lipid peroxidation

Lipid peroxidation is damaging due to the production of products which leads to the spread of free radical reactions. The potent function of lipids in the cellular system emphasizes the need to understand the consequences and mechanism of lipid peroxidation in the human body. Polyunsaturated fatty acids (PUFAs) are utilized as the substrate for the peroxidation of lipids due to the presence of active bis-allylic methylene groups. The hydrogen-carbon bonds on these methylene units have very low bond-dissociation energy, making the hydrogen atom to be abstracted easily in the radical reactions (Davies and Guo, 2014). It has been determined that the *a*-tocopherol is the most suitable antioxidant and protect the membrane from oxidation by reacting with the various lipid radicals generated in the peroxidation chain reaction of lipid. It carried out the removal of free radical intermediates and prevents the continuation of the propagation reaction (Mostafa Abd El-Aal, 2012). Antioxidants such as t-butyl hydroxy anisole (BHA), t-butylhydroxyl toluene (BHT), and vitamin E inhibited the Fe^2+^/ascorbate-induced peroxidation of lipids in the liver microsome of rats (Reddy and Lokesh, 1992). The natural agent, rosmarinic acid spontaneously penetrates the cell membrane to inhibit the peroxidation of lipids *in situ* as reported by Fadel et al. (2011).

### 5.4 Prevention of DNA damage

Oxidative stress is the major reason behind the majority of DNA damage in the case of human beings. Various factors are responsible for the production of free radicals and ROS. The major factors are smoking, junk foods/fried foods, restless life, and lifestyle change (Kaur et al., 2019). Antioxidant agents include external antioxidants and internal antioxidants that can be consumed through a diet to fulfil the natural need of the human body. These agents can scavenge free radicals and prevent further damage (Jamshidi et al., 2018). The fraction obtained from normal and transformed roots of *Rhaponticum carthamoides* provides DNA repair and antioxidant effect against oxidative stress-induced DNA damage in Chinese hamster ovary (CHO cells); (Skala et al., 2016). The methanolic fraction of *Tamarind indica, Adhatoda vasica, Centella asiatica, Pseudomugii furcatus, and Kocuria indica* protect against DNA damage. Trans-resveratrol and p-coumaric acid extracted from the ethanolic fraction of germinated peanut also protect against DNA damage (Limmongkon et al., 2019).

### 5.5 Prevention of protein modification

The oxidation of protein can be induced by several radical species such as hydroperoxyl, alkoxyl, peroxyl, •OH, O_2_^•-^, and by some non-radical species as well like OONO^−^, singlet oxygen, HOCl, O_3_, and H_2_O_2_. The reactive species oxidize the different types of amino acids present in the protein and result in the generation of the protein-protein cross linkage which further results in the denaturation, loss of protein functioning, loss of transport protein and receptor functioning, and loss of enzymatic activity. Hydroxyl radicals are reactive, they can further react with both inorganic and organic molecules like carbohydrates, lipids, proteins, and DNA and results in severe damage to cells (Phaniendra et al., 2015). Both biliverdin and bilirubin exhibited greater antioxidant activity than alpha-tocopherol against peroxynitrite and peroxyl-radical-induced protein oxidation in the brain microsome of rats *in vitro* (Mancuso et al., 2012).

### 5.6 Antioxidants’ protective effect against cancer

Chemoprevention of cancer is defined as the reverse process or inhibition of carcinogenesis by the administration of synthetic and natural agents as shown in Table 3. These agents have helped to understand the molecular and cellular levels of carcinogenesis. The chemopreventive agents can be divided into three wide categories.

(i) Those which prevent the generation of procarcinogens from the precursor components e.g., Vit. C, which avoids nitroso compounds formation (Landis-Piwowar and Iyer, 2014).
(ii) Blocking agents: These prevent the cancer casing compounds from interacting with the cellular target e.g., flavones, isothiocyanates, and phenols. Further, blocking agents are sub-categorized into three major categories: those which restrict the activation of carcinogen to its carcinogenic form; those which induce the enzyme system capable of carcinogen detoxification, and those which can react with cancer-causing species and prevent their reaction with the cellular targets (Steward and Brown, 2013; Landis-Piwowar and Iyer, 2014).
(iii) Suppressing agents: These restrict carcinogenesis by suppressing their activity e.g., protease inhibitors, sea salt, and retinoic acid (Usman et al., 2022).

**TABLE 3:** Role of antioxidants in the chemoprevention of other diseases.

Antioxidants	Diseases	Results/potential mechanism	References
Pre-clinical studies			
Resveratrol	Heart disease	↓ LDL,↓myocardial infarction risk, ↓coronary heart diseases	Conti et al. (2016)
Curcumin	Aging	↓ skin aging, ↓inflammation, ↓free radicals	Panahi et al. (2019)
	Alzheimer’s disease	↑Aβ disaggregation	Kamat et al. (2016)
Lupeol	Cataract	↑protection against nuclear opacity formation	Tewari et al. (2019)
Epigallocatechin-gallate	Alzheimer’s disease	↓Aβ, ↓amyloidosis	Kamat et al. (2016)
	Huntington’s disease	↓toxicity of mHTT and decline in motor function	
NAC, Beta-carotene, vitamin E	Parkinson disease	↓MPTP-directed neurotoxicity	
		↓loss of neurons	
Amygdaline	Neurodegenerative disease	↑Nrf2, ↓KEAP1, ↑antioxidant system in SH-SY5Y, N2a cells	Singh et al. (2021)
Hesperetin		↑ HO-1, ↑Nrf2 expression in BV2, HT22 cells	
Quercetin		↓neuroinflammation, ↑Nrf2/HO-1, ↓NF-kB	
Tenuigenin		↓neuroinflammation, ↑Keap1-Nrf2	
Delphinidin-3-glucoside	Coronary heart disease and Ischemia-reperfusion injury	↓caspase-3, ↓NOX2/NOX4	Chang et al. (2020)
		↓ROS, ↑autophagy, ↑AMPK/SIRT1	
Berberine	Hypertension	↑ AMPK/mTOR	
Ferulic acid	Heart failure	↑PI3K/Akt/mTOR, ↓ROS	
Gypenoside	Acute myocardial infarction	↑SOD, ↓free radicals	
Paeonol	Arrhythmia and coronary heart disease	↑SOD, ↓free radicals, ↓LPO	
p-Coumaric acid	Rheumatoid arthritis	↓TNF-α	Khanna et al. (2017)
Genistein	Rheumatoid arthritis	maintained a balance between Th2 and Th1	
		↓IL-4, ↓IFN-γ, ↓inflammation	
Curcumin	Rheumatoid arthritis	↓IL-6, ↓IL-1 in RA patient-derived fibroblast-like synoviocytes	
EGCG	Rheumatoid arthritis	↓Mcl-1, ↑apoptosis	
		↓MMP-3, ↓MMP-2, ↓MMP-1	
		↓cartilage and bone destruction	
Catechin	Cardiovascular disease	hypocholesterolemic effect	Velayutham et al. (2008)
		↓cholesterol esters, ↓low-density lipoprotein, ↓ plasma total cholestrol	
Lycopene	Sexual maturation	↑motility of sperm in broilers	Adewoyin et al. (2017)
Marjoram		↑ sperm and spermatogenic cells in the high fat diet	
		↑degenerative changes in seminiferous tubules	
Echinacoside		↓sperm damage which supports its capacity of profertility in SD rats, TM3, and LC-540 cells	Noh et al. (2020)
Soy isoflavones		↑sensitivity of insulin, ↑small leucine-rich proteoglycan	
Berberine-Rich fraction		↑quality of sperm, ↓inflammation, ↓oxidative stress related with male infertility	Saleh et al. (2018)
Zerumbone	Diabetes nephropathy	↓TGF-β1, ↓MCP-1, ↓fibronectin, ↓intercellular adhesion molecule-1 in the DN	Al-Waili et al. (2017)
Curcumin		Inhibits the expression of inflammatory genes by reversing caveolin Tyr phosphorylation that influenced the activation of Toll-like receptor 4	
Allicin		↓morphological alterations induced by diabetes in the kidney	
		↓triglyceride, ↓Scr, ↓BUN, ↓BGL,↓urine albumin	
		↓p-ERK1/2, ↓TGF-β1, ↓collagen I	
Rosmarinic acid		protective role against DN	
		↓ CTGF	
Quercetin, alpha-tocopherol, nifedipine, tetracycline	Ulcerogenesis	anti-ulcer effects in rat	Suzuki et al. (1998)
Sesamol		↓gastric mucosal myeloperoxidase	Elseweidy (2011)
		↓mucosal proinflammatory cytokines	
		↓oxide production, ↓lipid peroxidation, ↓nitric oxide	
Hesperidin	Depression	↓hyperglycaemia, ↑BDNF, ↑neurogenesis	Hritcu et al. (2017)
		↑ brain levels of monoamines in forced swimming test (FST) depression model	
Naringenin		↑glucocorticoid receptor (GR), ↑norepinephrine, ↑5-HT in tail suspension (TST) and open field test (OST)	
1,2,3,4,6-Penta-O-galloyl-beta-D-glucose (PGG)	Renal injury	↓excretion of calcium oxalate crystal	Deepika et al. (2013)
		↓ROS in EG-directed human primary renal epithelial cells (HRCs)	
Taurine		↓renal injury caused by oxidative stress, ↑GDH, ↑SOD	
		↓injury of mitochondrial membrane with lesser deposition of crystal in the kidney	
Lutein	Ischemic stroke	↓deleterious cerebral I/R outcomes and can be utilized as a treatment for stroke patients	Bahonar et al. (2017)
Essential oils from *Pinus roxburghii*	Tuberculosis	pollen dust and resin water used to treat tuberculosis	Verma et al. (2020)
Clinical studies			
Resveratrol	Superior thyroid artery from 59 old patients suffered with dyslipidemia and hypertension	↓ endothelial dysfunction *via* modulating the metabolism of nitric oxide and attenuation directed by vascular oxidative stress	Conti et al. (2016)
Curcumin	Central arterial compliance and arterial endothelial function in 32 post-menopausal women	↑improved the endothelial function	Akazawa et al. (2013)
Epigallocatechin-gallate	Multiple sclerosis in obese/overweight males	↑postprandial lipid oxidation that fuels the energy metabolism of muscles	Thielecke et al. (2010)
Quercetin	Hyperlipidemia in 400 patients	↓ total cholesterol, ↓triglycerides, ↓low-density lipoprotein, ↑increased high-density lipoprotein	Talirevic and Sehovic (2012)
	Type II diabetes mellitus in 24 patients	↓postprandial hyperglycemia within 30 min	Yi et al. (2021)
Genistein	Cardioprotective effect in 60 postmenopausal women	↓insulin level	Crisafulli et al. (2005)
		↓serum glucose level	
		↓deposition of fat in the heart and the blood vessels	
Berberine	Type II diabetes in 116 patients	↓triglycerides	Cicero and Sibel (2009)
		↓ low-density lipoprotein cholesterol	
Lycopene	20 patients with signs of gingivitis	↓gingivitis	Story et al. (2010)
		↓index of bleeding	
	17 asthmatic individuals	reduced influx of neutrophils to airways	
		↓ activity of neutrophil elastase that may improve the function of lungs in asthmatic individuals	
Vitamin E	2,102 individuals with the serum concentration of inflammatory cytokines	↓inflammatory cytokines	Asbaghi et al. (2020)
		↓TNF-α, ↓CRP, ↓IL-6	
Soy isoflavones	Bone health maintenance trial with 26 individuals	attenuated the bone resorption at the level of the femoral neck and lumbar spine	Gómez-Zorita et al. (2020)
	1,307 women at cardiovascular risk	↓triglycerides	
		↓total cholesterol concentration	
		↓oxidative stress markers	
		↓cardiovascular diseases risk	

Abbreviations and symbols: ↑increase, ↓decrease, CRP: C-reactive protein, LDL: Low-density lipoprotein; mHTT: mutant huntingtin; MPTP: 1-methyl-4-phenyl-1, 2,3,6-tetrahydropyridine; Nrf2: Nuclear factor erythroid 2-related factor 2; KEAP: Kelch-like ECH-associated protein; HO-1: Heme oxygenase-1; SH-SY5Y: neuroblastoma cell line; N2a: Neural crest-dericed cell line; BV-2: microglial cells; HT22: Hippocampal cell line; NOX2: NADPH, oxidase 2; NOX4: NADPH, oxidase 4; ROS: reactive oxygen species; AMPK: AMP-activated protein kinase; SIRT1: Sirtuin 1; mTOR: rapamycin; PI3K: Phosphoinositine 3-kinase; SOD: superoxide dismutase; TNF-α- Tumor necrosis factor alpha; Th2: T helper 2; Th1: T helper 1; IL-4: Interleukin 4; IFN-γ: interferon gamma; IL-6: Interleukin 6; IL-1; Interleukin 1; RA: rheumatoid arthritis; Mcl-1″ myceloid cell leukemia-1; MMP-3: Matrix metalloproteinase-3; MMP-2: Matrix metalloproteinase-2; MMP-1: Matrix metalloproteinase-1; TM3: leydig cells; LC-540: Cellosaurus cell line; TGF-β1: Transforming growth factor beta; MCP-1: Monocyte chemoattractant protein-1; DN: diabetes neuropathy; BUN: blodd urea nitrogen; BGL: blood glucose level; ERK1/2: Extracellular signal-regulated kinase ½; TGF-β1: Transforming growth factor-beta; CTGF: connective tissue growth factor; BDNF: Brain-derived neurotrophic factor; GDH: glutamate dehydrogenase.

Antioxidant defence such as the enzymes involved in the DNA damage repair cannot counteract all the oxidants and this results in damage which may lead to mutation and further contribute to carcinogenesis. Dietary antioxidants are ubiquitous and protect plants against oxidative assault and this property of them may be found to be useful in humans in terms of decreasing the risk of cancer. ROS are involved at all stages of tumor development, consequently, dietary agents are found to be protected throughout the stages of carcinogenesis. Citrus fruit is also reported to be beneficial in about 65% of investigational studies. The tumour-inhibitory effects imparted by these plant foods are due to the presence of various antioxidants such as carotenoids, polyphenolics, selenium, vitamin C, vitamin E, and provitamin A, respectively. It is determined that various constituents contribute to the overall protective effect (Bouayed and Bohn, 2010). Lung tumor risk has been reported to be decreased by the consumption of high vegetables and fruits in both retrospective epidemiological and prospective studies (Dela Cruz et al., 2011). Efficient inactivation of both endogenous and xenobiotics toxins results in the restriction of several cytotoxic events, and in the prevention of cellular integrity, which may lead to various diseases. The equilibrium between the phase II detoxifying enzyme and phase I carcinogen-activating enzyme is important to determine an individual’s risk of cancer disease (Wilkinson and Clapper, 1997). The contribution of various families of enzymes such as conjugation catalyzing transferase, hydrolases, peroxidases, reductases, dehydrogenases, and monooxygenases results in protection against different hazardous components like N-nitroso compounds, which are very different in their cellular defense mechanism. Some carcinogens act directly while others need activation (Rendic and Guengerich, 2012). The metabolism of the xenobiotic component is carried by many enzymes that are included in Phase II and I reactions (Jančová and Šiller, 2012). Phase I reactions include hydrolysis, reduction, hydroxylation, and oxidation, which results in water-soluble metabolites and further facilitate their excretion and conjugation. Cytochrome P450-dependent monooxygenase is the broadly studied phase I enzyme that is responsible for xenobiotic metabolism. These enzymes are encoded by the superfamily of CYP genes. In the NADPH monooxygenase system: P450 oxidoreductase transfer the electrons from NADPH to P450 and results in the formation of ferrocytochrome P450 which carried out molecular oxygen activation and one of the atoms of oxygen is added to the substrate molecule (Riddick et al., 2013). Other enzymes of phase I include: reductase, aromatase, dehydrogenase, monoamine oxidase, hydrolase, lipoxygenase, cyclooxygenase, and monooxygenase. The products of phase I reactions act as a substrate for the phase II enzymes but some of the xenobiotics are conjugated directly bypassing the metabolism of phase I. Cytochrome b5 is one of the competitive inhibitors of CytP450 phosphorylation by protein kinase. Thus, b5 has a key role in the activity of cytP450. Mixed function oxidase is the flavin comprising monooxygenase present in the ER and contains one FAD molecule. The endogenous substrate for this is cysteamine and it can oxidize the nucleophilic sulphur and nitrogen atom (Porter, 2002). Detoxification enzymes of phase II compete with the activating enzymes of phase I to restrict the generation of electrophiles and catalyze the conversion of these electrophilic molecules to inactivate conjugates, making them more soluble in water and more easily get excreted from the body. Some of the conjugating reactions are glutathione conjugation, sulfation, and glucuronidation, respectively (Hodges and Minich, 2015).

### 5.7 Antioxidant’s protective effects against other diseases

The consumption of antioxidants through vegetables and fruits that are determined to be the better source of antioxidants helping in the treatment of cardiovascular diseases. These antioxidants are also utilized in the prevention of neurodegenerative diseases such as Parkinson’s and Alzheimer’s disease. Excessive production of reactive species leads to various pathological diseases such as ulcerogenic, depression, cardiovascular disorders, and rheumatoid arthritis. Antioxidants have been considered to play an important role in the prevention of these diseases. It has been found that antioxidants have a better potential for the treatment of problems related to sexual maturity, male infertility, and nephrolithiasis (Sindhi et al., 2013), (Table 3). Despite of various outcomes reported *in vitro* and in animal models, some studies are concentrated to humans and the results obtained are represented below in Table.

## 6 Therapeutic limitations

Natural antioxidant agents have many modes of action and can be useful in preventing diseases without side effects (Chaudhary and Janmeda, 2022b). The availability of antioxidants should be regulated by a prescription from a healthcare professional. Consumers should be advised about the health benefits of antioxidants and be encouraged to eat foods rich in vegetable oils, nuts, seeds, leafy vegetables and fresh fruits, which are the main sources of antioxidants. The main therapeutic limitation is the excessive consumption of antioxidant supplements that can cause side effects because in high concentrations antioxidants can act as pro-oxidants. There is also a significant difference between taking antioxidants from food and administering an isolated substance as a supplement. Many substances that show beneficial effects in the laboratory do not work when they are introduced into the human body. Many antioxidants do not have good bioavailability. The concentration of antioxidants such as polyphenols is sometimes so low in the blood that no significant effect is observed (Del Rio et al., 2013).

## 7 Conclusion and future perspectives

Free radicals-directed oxidative stress is known to be harmful to the health of a human being. Various experimental studies determine that free radicals contribute towards the progression and inhibition of various pathologies, ranging from cardiovascular disease to cancer. Antioxidants can counteract oxidative stress and mitigate all the effects on human health. These compounds gained a lot of attention from the field of biomedical research as they showed a better degree of efficacy in terms of the cure and prevention of various diseases. Synthetic antioxidants are found to be detrimental to the health of an organism. Therefore, the search for a non-toxic and natural compound with greater antioxidant activity has increased in the last few years. Through the literature survey, we can conclude that oxidative stress should be exploited as a tool for the treatment when and if we would be able to understand the fine-tuning of this phenomenon inside a living body. Newer approaches that utilise modern technology and collaborative research in combination with established conventional health practices can be used in near future for the improvement of health status, especially among individuals who do not have access to costlier therapeutic drugs.

## Figures and Tables

**Table:** 

**NO**	Nitric oxide
**OH**	Hydroxyl
**^1^O_2_**	Singlet oxygen
**AP-1**	Activator protein
**BHA**	Butylated hydroxyanisole
**BHT**	Butylated hydrox ytoulene
**BNDF**	Brain-derived neutrophic factor
**CAFs**	Carcinoma associated fibroblast
**CAT**	Catalase
**COX2**	Cyclooxygenase 2
**Cu**	Copper
**DNA**	Deoxyribonucleic acid
**EMT**	Epithelial-mesenchymal transition
**G6PD**	Glucose-6-phosphate dehydrogenase
**GPx**	Glutathione peroxidase
**GSH**	Glutathione
**H_2_O_2_**	Hydrogen peroxide
**HCC**	Hepatocellular carcinoma
**HDL**	High-density lipoprotein
**HIF-1α**	Hypoxia-inducible factor
**HOCl**	Hypochlorous acid
**IL-6**	Interleukin-6
**iNOS**	inducible nitric oxide synthase
**LDL**	Low-density lipoprotein
**LOX**	Lipooxygenase
**LPS**	Lipopolysaccharides
**MAPK**	Mitogen-activated protein kinase
**MMP9**	Metalloproteinase 9
**MPO**	Myeloperoxidase
**NADPH**	Nicotinamide adenine dinucleotide phosphate
**NO**	Nitric oxide
**O_2_^.-^**	Superoxide
**ONOO^−^**	Peroxynitrite
**-OR**	Alkoxy
**POAF**	Post-operative atrial fibrillation
**PPAR-γ**	Peroxisome proliferated activated receptor gamma
**PPP**	Pentose phosphate pathway
**PTEN**	Phosphatase and tensin homolog
**PUFAs**	Polyunsaturated fatty acids
**RA**	Rheumatoid arthritis
**RNA**	Ribonucleic acid
**RNS**	Reactive nitrogen species
**ROO**	Peroxyl
**ROS**	Reactive oxygen species
**SOD**	Superoxide dismutase
**STATs**	Signal transducer and activator of transcription
**TLR**	Toll-like receptor
**TNF-α**	Tumor necrosis factor alpha
**VEGF**	Vascular endothelial growth factor
**XDO**	Xanthine dehydrogenase
**XO**	Xanthine oxidase
**Zn**	Zinc
